# Hemorrhage into the bile duct after endoscopic ultrasound-guided fine needle aspiration for pancreatic cancer

**DOI:** 10.1055/a-2313-3991

**Published:** 2024-05-17

**Authors:** Kazuya Miyaguchi, Suguru Mizuno, Satoshi Mochida

**Affiliations:** 1 539165 Department of Gastroenterology and Hepatology, Faculty of Medicine, Saitama Medical University, Saitama, Japan

A 71-year-old man presented to our department with an elevated serum amylase level of 695 IU/L. Computed tomography revealed a cystic lesion measuring 26 mm in diameter in the pancreatic head ( Fig. 1 ). Endoscopic ultrasound (EUS) identified a hypoechoic tumor measuring 15 mm in diameter adjacent to this multilocular cyst. The common bile duct (CBD) was compressed by the tumor without proximal dilation ( Fig. 2 ). We performed EUS-guided fine needle aspiration (EUS-FNA) using a 22-gauge Franseen needle from the duodenal bulb in the long scope position. After three punctures, Doppler imaging revealed a turbulent flow signal in the CBD ( Fig. 3 ). Subsequently, we confirmed bleeding from the papilla endoscopically ( Fig. 4 ). Additionally, hyperechoic clots in the gallbladder were observed endosonographically ( Fig. 5 ). The patient remained stable, with a gradual reduction in the turbulent flow signal. Thereafter, we performed endoscopic biliary drainage using a 5-Fr nasobiliary catheter to monitor hemobilia and found no rebleeding **.** The pathological diagnosis was adenocarcinoma. ( Video 1 ).

**Fig. 1:**
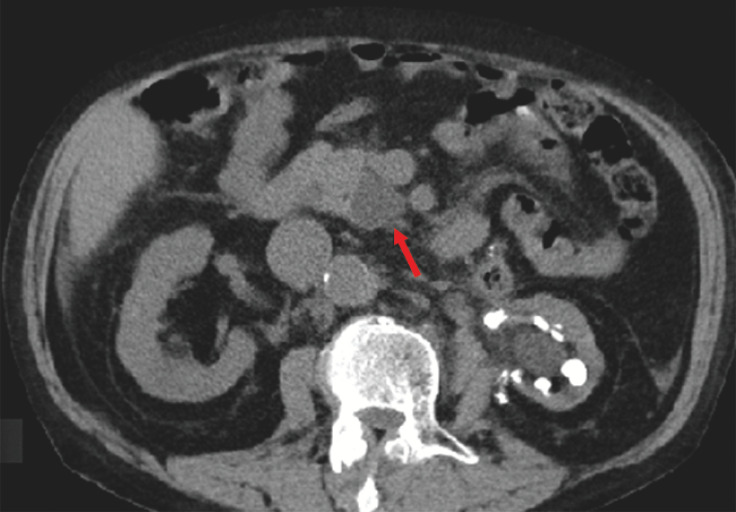
Computed tomography shows a cystic lesion in the pancreatic head (red arrow).

**Fig. 2:**
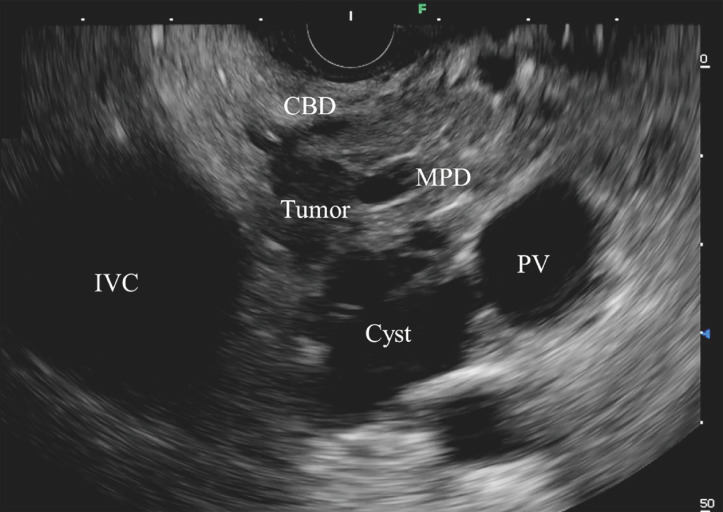
Endoscopic ultrasound shows a hypoechoic tumor adjacent to the multilocular cyst.

**Fig. 3:**
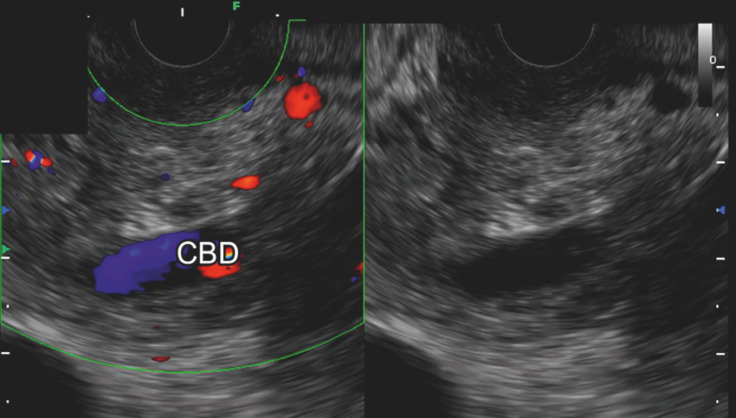
A turbulent flow signal was observed in the common bile duct after endoscopic ultrasound-guided fine needle aspiration.

**Fig. 4:**
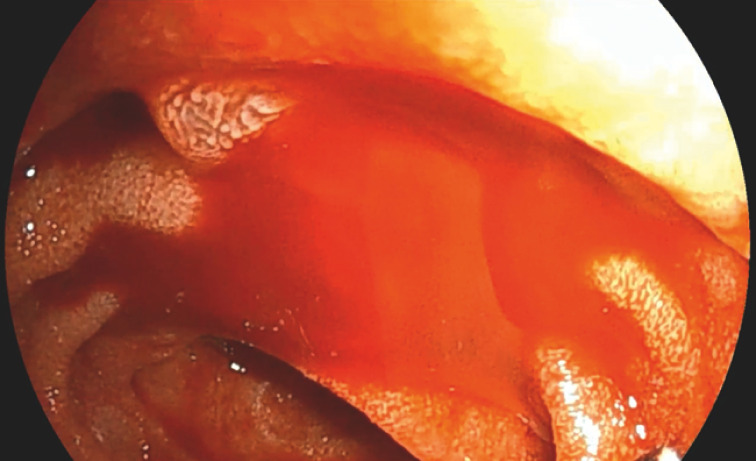
Bleeding from the papilla was observed endoscopically.

**Fig. 5:**
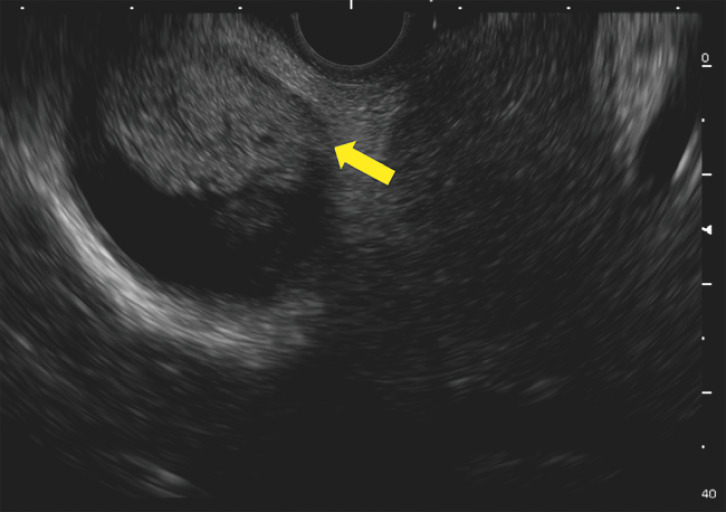
Hyperechoic clots were seen in the gallbladder (yellow arrow).

Hemorrhage into the bile duct was detected as a turbulent flow signal by Doppler imaging after endoscopic ultrasound-guided fine needle aspiration for a pancreatic head tumor.Video 1

The incidence of iatrogenic hemobilia is reportedly increasing 1 . However, hemorrhage is a rare complication during EUS-FNA 2 , and only two case reports of hemobilia after EUS-FNA have been documented 3 4 . In our case, an impressive video of the turbulent flow signal by Doppler imaging was captured as a sign of hemobilia. Fortunately, hemobilia stopped spontaneously in this case. If the bleeding was severe, interventional radiology might have been needed. Despite taking precautions to determine a puncture route to avoid injuring intervening vessels and organs by B-mode and Doppler imaging, small arteries adjacent to the CBD might remain undetectable due to scope compression. To ensure early detection of adverse events following EUS-FNA, it is essential to assess for hemorrhage both endosonographically and endoscopically.

Endoscopy_UCTN_Code_CPL_1AL_2AD

## Notes

Endoscopy E-Videos https://eref.thieme.de/e-videos
*E-Videos* is an open access online section of the journal *Endoscopy* , reporting on interesting cases and new techniques in gastroenterological endoscopy. All papers include a high-quality video and are published with a Creative Commons CC-BY license. Endoscopy E-Videos qualify for HINARI discounts and waivers and eligibility is automatically checked during the submission process. We grant 100% waivers to articles whose corresponding authors are based in Group A countries and 50% waivers to those who are based in Group B countries as classified by Research4Life (see: https://www.research4life.org/access/eligibility/ ). This section has its own submission website at https://mc.manuscriptcentral.com/e-videos .

## References

[1] Zhornitskiy A Berr R Han JY Hemobilia: Historical overview, clinical update, and current practice Liver Int 2019 39 1378 1388 30932305 10.1111/liv.14111

[2] Eloubeidi MA Tamhane A Varadarajulu S Frequency of major complications after EUS-guided FNA of solid pancreatic masses: a prospective evaluation Gastrointest Endosc 2006 63 622 629 10.1016/j.gie.2005.05.024 16564863

[3] Kawakubo K Isayama H Takahara N Hemobilia as a rare complication after endoscopic ultrasound-guided fine-needle aspiration for hilar cholangiocarcinoma Endoscopy 2011 43 E334 335 10.1055/s-0030-1256783 22020713

[4] Horiuchi T Shibata Y Shinomiya W Biliary tract bleeding with obstructive jaundice after endoscopic ultrasound-guided fine-needle aspiration of a pancreatic head tumor Clin J Gastroenterol 2020 13 116 119 31165459 10.1007/s12328-019-01000-x

